# Application of Patterson-function direct methods to materials characterization

**DOI:** 10.1107/S2052252514017606

**Published:** 2014-08-29

**Authors:** Jordi Rius

**Affiliations:** a Institut de Ciència de Materials de Barcelona, CSIC, Campus de la UAB, Bellaterra, Catalonia 08193, Spain

**Keywords:** direct methods, PFDM, δ recycling, *S*-FFT, *S*-TF, cluster-based DM, powder diffraction, *ab initio* structure solution, precession electron diffraction, electron diffraction tomography

## Abstract

The aim of this article is a general description of the so-called Patterson-function direct methods (PFDM), from their origin to their present state. It covers a 20-year period of methodological contributions to crystal structure solution, most of them published in *Acta Crystallographica Section A*. The common feature of these variants of direct methods is the introduction of the experimental intensities in the form of the Fourier coefficients of origin-free Patterson-type functions, which allows the active use of both strong and weak reflections. The different optimization algorithms are discussed and their performances compared. This review focuses not only on those PFDM applications related to powder diffraction data but also on some recent results obtained with electron diffraction tomography data.

## Abstract 2

Patterson-function direct methods are chronologically reviewed and their applications to powder and electron diffraction are described.

## 1. Introduction   

Patterson-function direct methods (PFDM) are those direct methods (DM) extracting the phase information directly from the non-origin part of the experimental Patterson-type function. Although the first method of this category was described quite early by Rius (1993 ▶), the fact that PFDM lie halfway between traditional DM and Patterson deconvolution methods is surely one of the reasons why they are not as popular as other structure solution methods. The aim of the present article is to provide a comprehensive description of the advances in PFDM during the last 20 years and, at the same time, to introduce a rational classification and consistent nomenclature for their different variants. This clarification should help to increase their dissemination and to promote their wider use. PFDM are extremely simple both theoretically and computationally, and are especially well suited to such problems where not only the strong but also the weak intensities are accessible from the experiment. This comprises most applications to materials science dealing with crystalline matter. Although the present contribution focuses on the phasing of powder X-ray diffraction (PD) and electron diffraction (ED) data of inorganic materials, most of the results can be applied to any kind of material.

## 2. Patterson-function direct methods based on ρ^2^   

Before starting with the description of PFDM, a short introduction to the quantities involved in their definition is in order. In this review, for simplicity, an equal-atom crystal structure belonging to space group *P*1 with *N* atoms in the unit cell is assumed. In addition, bold letters denote complex or vector quantities, while standard text indicates the corresponding moduli (amplitudes). The observed quantities are the normalized *E* amplitudes, *i.e.* the *F* structure factor amplitudes corrected for fall-off in sin θ/λ (mainly due to the atomic form factor evolution and to the atomic thermal vibration; θ is half the diffraction angle and λ is the incident wavelength). For an arbitrary **H** reflection, the corresponding *E* amplitude is given by
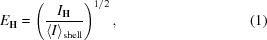
and can be derived from the measured intensity *I* and the average intensity in the corresponding reciprocal-space shell, 〈*I*〉_shell_. A Fourier synthesis with the ***E*** values as Fourier coefficients yields the sharpened electron density distribution (ρ) of the crystal. The ***E*** values are complex quantities with their amplitudes known but with their associated phases, ϕ, lost during the diffraction experiment. Especially relevant for the development of DM was the derivation of the probability distribution of the structure factor (s.f.) amplitudes by Wilson (1949 ▶), in which he assumed that the atomic positions were random variables with uniform distribution throughout the unit cell. Written in terms of *E*, the probability distribution is (for *P*1 symmetry)
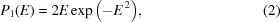
with the moments of *P*_1_(*E*) being 〈*E* ^2^〉 = 1 and 〈*E*〉 = 0.9, and with associated variance
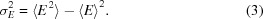
The basic assumption made, *i.e.* that all points in the unit cell have the same probability of hosting an atom, constitutes the ‘randomness’ condition. An important property of equation (2) is that *P*_1_(*E*) is independent of the number of atoms *N* in the unit cell. Physically, the *E* values represent the amplitudes of a hypothetical unit cell consisting of point atoms with a scattering power equal to 1/(*N*)^1/2^. Consequently, the amplitude of the s.f. of ρ^2^, **G** = *G*exp(*i*ψ), is given by the simple relationship
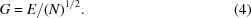
In view of equation (4), *G* can also be considered experimentally accessible, so that both *E* and *G* can be used interchangeably.

### 2.1. The calculated structure factor amplitudes   

If Φ represents the subset of refined phases belonging to the **h** reflections with large *E* values, then the *G*(Φ) amplitudes of the squared structure can be expressed in terms of the Fourier coefficients *E*exp(*i*ϕ) by Fourier transforming ρ^2^(Φ) = ρ(Φ)·ρ(Φ) and posterior multiplication with exp(*i*ψ_−**H**_), *i.e.* by means of the summation
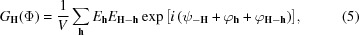
with
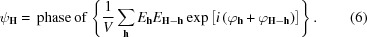
The equal-peak condition is implicit in the squaring operation, whereas positivity is forced if ψ**_h_** and ϕ**_h_** are equated (only for strong reflections), *i.e.* by assuming that ρ^2^(Φ) is directly proportional to ρ(Φ) (Sayre, 1952 ▶). However, the randomness condition is not included in the squaring operation and hence will depend on each particular phasing method. Traditional DM procedures were not especially robust regarding this condition, as proved by the frequently occurring uranium-atom solution. For a long time, this solution represented a serious DM limitation and it is characterized by the appearance of an outstanding strong peak in the Fourier map. Although the equal-peak and positivity conditions are not violated, this solution is clearly wrong.

### 2.2. The origin-free modulus sum function (*S*_*M*_)   

A Fourier synthesis with the *G* amplitudes as coefficients yields the modulus synthesis (*M*) of ρ^2^, which is a Patterson-like synthesis with a dominant origin peak. Similarly, a synthesis with coefficients *G* ^2^ yields the true Patterson function, *P*, of ρ^2^. If the strong origin peak is removed from *M*, the non-origin peaks become dominant. If *M*′ denotes *M* with no origin peak, the phasing residual
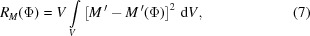
will measure, as a function of Φ, the discrepancy between observed and calculated *M*′ over the whole unit cell. The Fourier coefficients of *M*′(Φ) are *G*_**H**_(Φ) − 〈*G*(Φ)〉, which can be derived from equation (5). By applying the Fourier theory, *R*_*M*_(Φ) can be worked out to (*Appendix A* [App appa])
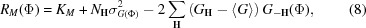
where *G*_**H**_ − 〈*G*〉 are the Fourier coefficients of the observed *M*′. The first term, *K_M_*, is a phase-independent quantity. The second term, 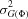, is the variance of the probability distribution of the *G*(Φ) amplitudes that can also be assumed to be phase-independent for Φ sets satisfying the equal-atom and randomness conditions (irrespective of the correctness of Φ). In general, these two assumptions are valid because the equal-atom condition is implicit in the squaring operation and because, by only considering the non-origin peaks of the modulus function (which correspond to interatomic vectors ranging over the whole unit cell), randomness is favoured. Consequently, minimizing *R* is essentially equivalent to maximizing the third term of equation (8), the so-called origin-free modulus sum function
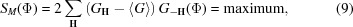
which, after replacing *G*_−**H**_(Φ) by equation (5), becomes
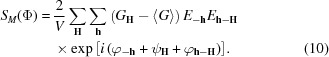
The *S*_*M*_ sum function [originally called *Z_R_* in Rius (1993 ▶)] represents one of the last advances in reciprocal-space DM. Since the true Φ corresponds to a maximum in *S*_*M*_(Φ), a simple method for its maximization is needed. For this purpose, the order of the double summation in equation (10) is changed, so that it becomes
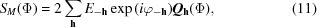
with ***Q***_**h**_(Φ) being
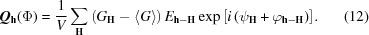
If 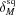 denotes the Fourier synthesis with coefficients (*G*_**H**_ − 〈*G*〉)exp(*i*ψ_**H**_), equation (12) can also be expressed as the Fourier coefficient of the 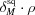 product function, *i.e.*

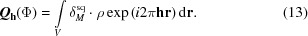
By following Debaerdemaeker *et al.* (1985 ▶), the maximum of a functional like *S*_*M*_ can be found by solving the condition for an extremum, *i.e.* by making
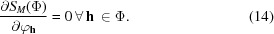
If this condition is applied to equation (11), a tangent formula (TF) is obtained which provides the new phase estimates
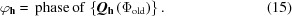
Depending on whether ***Q*_h_** is expressed in terms of the ψ and ϕ phases [equation (12)] or as a function of 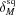 [equation (13)], two different optimization algorithms result: (i) the sequential *S*_*M*_ tangent formula (*S*-TF algorithm) based on phase invariants, and (ii) the parallel *S*_*M*_ tangent formula (*S*-FFT algorithm) based on Fourier transforms. For simplicity, the subscripts of *S* (*i.e. M* or *P*) have been omitted from the general algorithm designation.

In equation (9), the experimental quantities are the amplitudes. However, for certain applications it can be desirable to work directly with intensities. That this is feasible can be easily shown by considering the physical meaning of *S*_*M*_(Φ) in equation (9), which corresponds to the integral
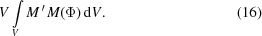
It is known that the principal differences between Patterson and modulus functions are the relative heights between origin and non-origin peaks. If the origin peaks are suppressed, the resulting *P*′ and *M*′ functions may be regarded as proportional by a factor close to two (Rius, 2012*b* ▶). Consequently, maximizing equation (16) is equivalent to maximizing the integral
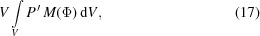
or in terms of the respective Fourier coefficients
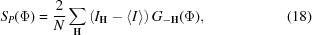
since 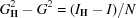. Notice that the ***Q***_**h**_(Φ) expressions, equations (12) and (13), are also valid for *S*_*P*_ simply by replacing (*G*_**H**_ − *G*) by 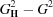 and 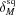 by 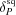, respectively. *S*_*P*_ is particularly useful for powder diffraction (PD), because working with experimental intensities simplifies the manipulation of overlapped intensities.

### 2.3. Phase refinement algorithms based on *S*   

#### 2.3.1. Sequential application of the tangent formula with phase invariants (*S*-TF algorithm)   

One possibility of maximizing the *S*_*M*_ sum function is by means of the iterative application of the tangent formula of equation (15) with ***Q***_**h**_ given in terms of the phases of equation (12). In practice, the **H** summation (involving all reflections) is split into two separate sums: the **K** one (strong reflections) and the **L** one (weak reflections). Only for the **K** sum is no distinction made between the ψ and ϕ phases. This causes the three summands having phase invariants ϕ**_−h_** + ϕ**_K_** + ϕ**_h−K_**, ϕ**_K_** + ϕ**_−h_** + ϕ**_h−K_** and ϕ**_−h_** + ϕ**_h−K_** + ϕ**_K_** to have the same Φ3**_hK_** phase sum. Consequently, they can be replaced in the sum by
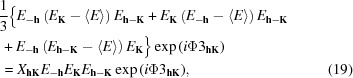
where 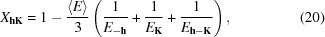
and ***Q***_**h**_ becomes
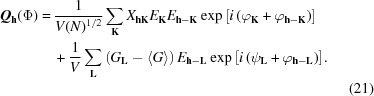
According to equation (14), phases refined with the tangent formula of equation (15) will lead to an extremum in *S*_*M*_, *i.e.* to a large maximum or a large minimum. However, since for strong reflections *S*-TF makes ψ and ϕ equal, only positive solutions are possible. In the *S*-TF algorithm the TF is applied in sequential mode. This means that, once a new ϕ**_h_** estimate is calculated with equation (15), its value is immediately replaced in Φ. The updated Φ is then used to compute the phase estimate of the next reflection. This process is repeated until all **h** reflections in Φ have been treated and no significant phase variations are observed. Before starting a new iteration cycle, the ψ phases of the weak reflections are updated using equation (6) (for strong reflections this is done automatically, because ψ and ϕ are considered equal). This means that the *S*-TF algorithm is essentially a two-stage process in which the estimates of the ϕ and ψ phases are updated alternately.

*S*-TF is very effective, easy to apply, and makes no use of any Fourier synthesis. It is ideal for solving small-molecule crystal structures. However, for crystal structures with a large number of atoms in the asymmetric unit (>500 atoms) the total number of terms in the **L** summation becomes prohibitive. The introduction of a higher cut-off value, *E*_min_, for large *E* values reduces the number of invariant terms at the cost of lowering the accuracy of the calculated *G*(Φ). To check the efficiency of the *S*-TF algorithm, the phasing power of *S*-TF was compared with the power of the traditional TF (Karle & Hauptman, 1956 ▶), strengthened with information on the most reliable negative quartets. For crystal structures with no fixed space group origin, the success rate of *S*-TF was one order of magnitude higher (Table 1 ▶) (Rius *et al.*, 1995 ▶; Sheldrick, 1990 ▶).

**Figure 1:**
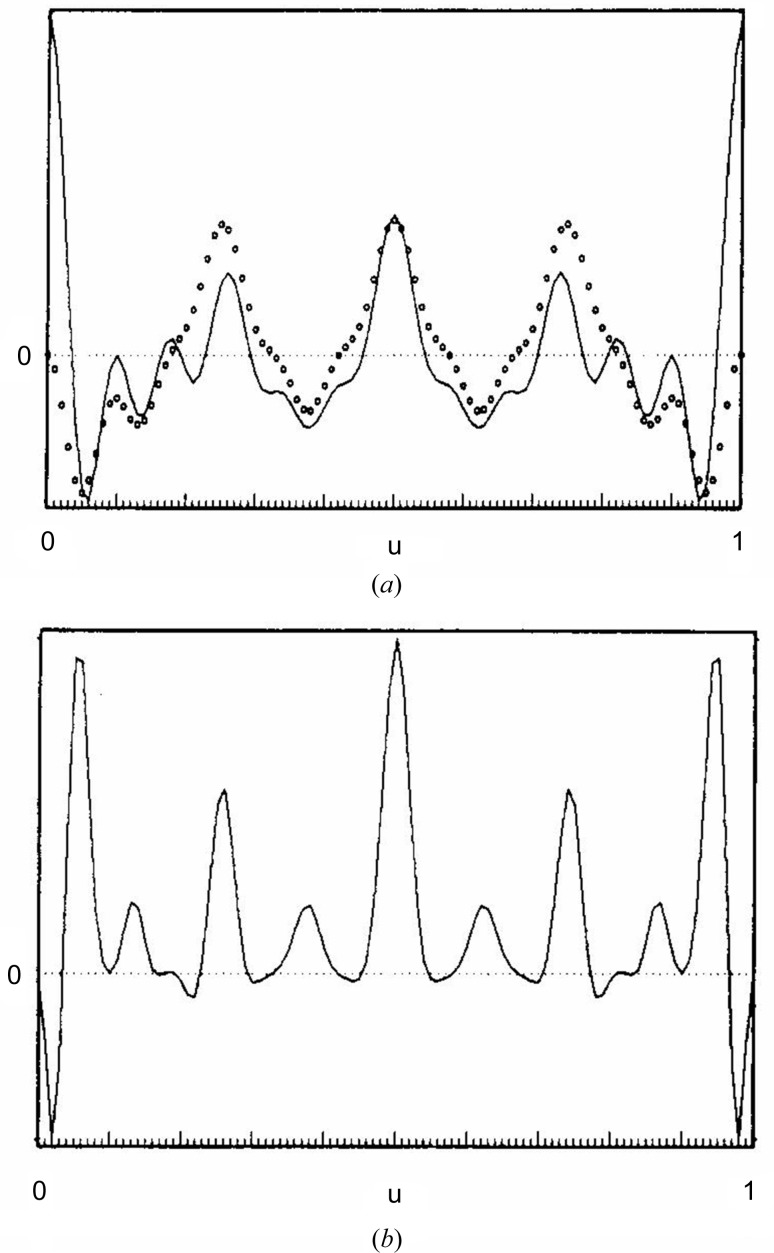
Physical interpretation of the *S*_*M*_ function of equation (9). (*a*) Calculated modulus function *M* expressed in terms of the true Φ (line) and corresponding observed origin-free modulus function *M*′ (dots). (*b*) The *M*′*M*(Φ) product function. Since *M*′ has no origin peak, the peaks of the product function are evenly distributed along the unit cell (all interatomic peaks contribute). If the origin peak were present, it would play a dominant role in the product function, so that a solution of Φ giving rise to a single very strong peak in the *E* map would be a positive maximum of equation (23).

In retrospect, one possible explanation for the late discovery of PFDM can be found in the leading role played by the integral
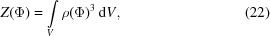
in the development of DM (Cochran, 1955 ▶). It can easily be shown that this integral is closely related to the sum function
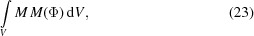
since both give similar values (in both expressions the weak *E* values play no role). However, conceptually, both are completely different. While further progress from *Z* is not evident, the sum function of equation (23) can evolve to the *S_M_* function of equation (11). The reason why the latter represents an improvement is explained intuitively in Fig. 1 ▶.

At the beginning of this article it was stated that the first PFDM was published in 1993. This is only partially true, because the most reliable negative quartets (Schenk, 1973 ▶; Giacovazzo, 1976 ▶) can also be derived from Patterson-function arguments, *i.e.* by expressing the integral
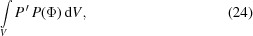
as a function of the Φ phases. Since the non-origin parts of the modulus and Patterson functions of ρ^2^ can be considered proportional, maximizing the integral of equation (24) is equivalent to maximizing equation (16), so that in this case ***Q*_h_** takes the form (Rius, 1997 ▶)
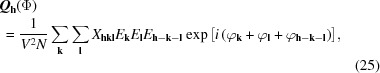
where 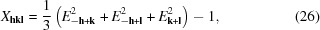
and **h**, **k**, **l** and **−h**, **−k**, **−l** belong to the subset of strong reflections and *X* **_hkl_** involves both strong and weak reflections. Notice that *X* **_hkl_** becomes clearly negative only when the three squared amplitudes in equation (26) correspond to weak reflections. One immediate difference between equations (21) and (25) is that the estimation of a new phase with equation (25) requires the lengthy calculation of a double summation. In addition, manipulation of the mixed terms in *X* **_hkl_** is far from trivial.

One year after the introduction of *S*-TF, the first paper on the Shake-and-Bake phasing method was published (DeTitta *et al.*, 1994 ▶). It represented a radical change in DM philosophy, since it combined phase refinement in reciprocal space with Fourier filtering, thus exploiting the considerable computing power already available at that moment. In this way the trend towards the uranium-atom solution of traditional reciprocal DM was compensated by the periodic reintroduction of randomness during the direct-space stage by picking up the *N* largest Fourier peaks. This new way of preserving randomness did not rely on the weak reflections. This circumstance proved particularly useful in the solution of *e.g.* anomalous scatterer substructures in macromolecules. However, when weak reflections are available (as in PD or ED applications), PFDM are highly competitive. As will be shown in the next section, PFDM can also be optimized entirely in direct space (*S*-FFT algorithm), so that if necessary they can also be strengthened by Fourier filtering.

**Figure 2:**
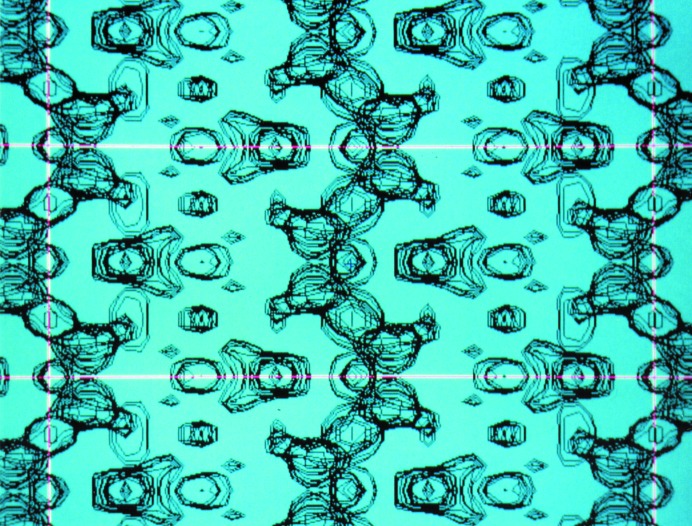
RUB-15 layered silicate solved by assuming *Ibam* symmetry (*a* = 27.91, *b* = 8.41, *c* = 11.52 Å). Superimposed Fourier sections (*y*/*b* = 0.00–0.45) show the silicate sheets normal to [001]. The phases were obtained by *S*-TF from laboratory PD data at moderate resolution (*d* ≥ 2.2 Å). At this resolution, the tetrahedral Si units appear as spheres. The tetra­methyl­ammonium molecules are placed on the mirror planes.

Between the publications of the *S*-TF and *S*-FFT algorithms, 14 years elapsed. During this period some new PD applications of *S*-TF were explored. One example is the crystal structure solution of the layered zeolite-like silicate RUB-15 of the formula TMA_8_[Si_24_O_52_(OH)_4_]·20H_2_O from laboratory PD data (TMA = tetramethylammonium cation; Oberhagemann *et al.*, 1996 ▶). At that time it was still a common belief that DM needed intensity data at atomic resolution to be successful. However, the determination of RUB-15 demonstrated that, if the electron density of the main building elements can be roughly approximated at moderate resolution to broad spherical peaks (*d*_min_ ≃ 2 Å), DM will work. In RUB-15, both the SiO_4_ and TMA tetrahedra were handled in this way (Fig. 2 ▶). This approach allowed the solution of a series of layered zeolite-like compounds at moderate resolution in collaboration with the Institute for Mineralogy of the Ruhr University Bochum (Gies *et al.*, 1998 ▶).

Another important result was the demonstration that *S*-TF can be applied to PD data of hemihedral compounds (Rius *et al.*, 1999 ▶). This was confirmed by solving the crystal structures of: (i) the CAH10 binding phase in high-alumina cement (space group *P*6_3_/*m*; Guirado *et al.*, 1998 ▶), and (ii) aerinite, a natural blue pigment employed in some Catalan romanesque mural paintings (space group *P*3*c*1; Rius *et al.*, 2004 ▶). Both crystal structures had resisted multiple attempts at solution worldwide.

In the literature there are various methods of combining information from multiple PD patterns, *e.g.* by making use of the anisotropic thermal expansion of a material (Shankland *et al.*, 1997 ▶). In the particular case of zeolites, the information contained in the powder patterns of the as-synthesized and calcined forms can easily be exploited in a two-stage procedure (Rius-Palleiro *et al.*, 2005 ▶). In the first stage, the template molecule is located by combining isomorphous replacement with *S*-TF at very low resolution (*d*_min_ ≃ 3.2 Å), whereas in the second stage, the framework atoms are found by again applying the *S*-TF algorithm but now strengthened with the information coming from the located template molecules (*d*_min_ ≃ 2.21 Å). This procedure was applied to the solution of the ITQ-32 zeolite (Cantín *et al.*, 2005 ▶).

All the *S*-TF applications described so far use the resolved reflections exclusively (except for hemihedral symmetries, where the intensities of systematically overlapping reflections were equidistributed and treated as resolved), so that strictly speaking these may be regarded as single-crystal applications. However, the solution of the triclinic crystal structure of tinticite, a partially disordered phosphate mineral, required a more sophisticated *S*-TF procedure in which not only the phases were refined but also the estimated intensities of the severely overlapped peaks (*d*_min_ ≃ 2.3 Å) (Rius, Torrelles *et al.*, 2000 ▶). In the best *E* map, the broad spherical peaks corresponding to the [Fe^III^O_6_] octahedra and to the phosphate tetrahedra (the latter with partial occupancies) showed up clearly (Rius, Loüer *et al.*, 2000 ▶). In spite of this success, the refinement often had stability problems, undoubtedly due to the inaccurate intensity estimation of overlapping reflections from a limited number of invariant terms.

#### 2.3.2. Parallel application of the tangent formula *via* Fourier transforms (*S*-FFT algorithm)   

**Figure 3:**
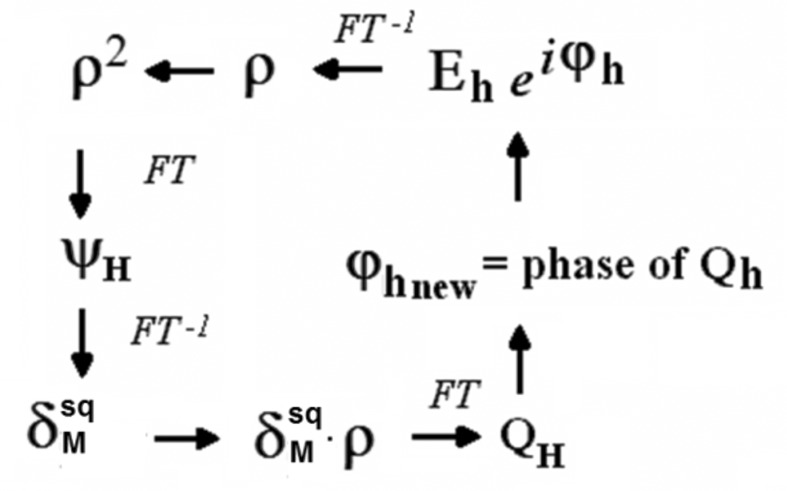
Iterative *S*-FFT phase refinement procedure. The initial phase values (ϕ) are combined with the experimental *E* amplitudes to give the initial ρ values (upper right corner). The ψ phases are obtained by Fourier transforming ρ^2^. Combination of ψ with the experimental (*G* − 〈*G*〉) gives the Fourier coefficients of the 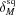 synthesis. The new structure factor estimates *Q* are obtained by Fourier transforming the δ·ρ product function.

Historically, the development of the *S*-FFT algorithm is related to the 23rd European Crystallographic Meeting in Leuven (2006). On the occasion of that meeting, Professor Baerlocher (ETH, Zurich) showed to the author the potential of charge-flipping when applied to PD (Baerlocher *et al.*, 2007 ▶; Palatinus, 2013 ▶). Spurred on by this result, the rationale behind charge-flipping was sought. During this search it was found that *S*_*M*_ can also be maximized by Fourier methods (Rius *et al.*, 2007 ▶). In contrast with the *S*-TF algorithm, where the new ϕ_**h**_ are estimated sequentially, the *S*-FFT algorithm determines the new ϕ_**h**_ (the Fourier transforms of 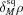) in parallel, *i.e.* from a unique Φ_old_. A second important difference between the two algorithms is that in *S*-FFT the alternating update of the ψ and ϕ phases is done in completely separate stages (no explicit use is made of the equality between ψ and ϕ). The two stages of one iteration cycle are (Fig. 3 ▶) 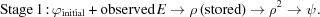


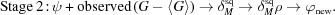
Since the TF refinement leads to an extremum in *S*_*M*_ and the condition ψ**_h_** = ϕ**_h_** is not applied during the refinement, it can produce either ρ or −ρ as valid solutions when starting from random phases.

The algorithm works quite well with single-crystal data of small- and medium-sized structures at atomic resolution. The stability of the algorithm is reflected in the fact that no electron-density modification is required after each refinement cycle, *e.g.* there is no need to suppress negative values or for periodic reintroduction of randomness in Φ by selecting the *N* highest peaks in the Fourier map (and posterior recalculation of Φ from these peaks). It is clear that, for small crystal structures, the phase refinement efficiencies of *S*-TF and *S*-FFT must be similar. Table 2 ▶ compares the respective efficiencies for a selection of representative compounds.

#### 2.3.3. The *S*-FFT algorithm extended to non-positive definite ρ   

**Figure 4:**
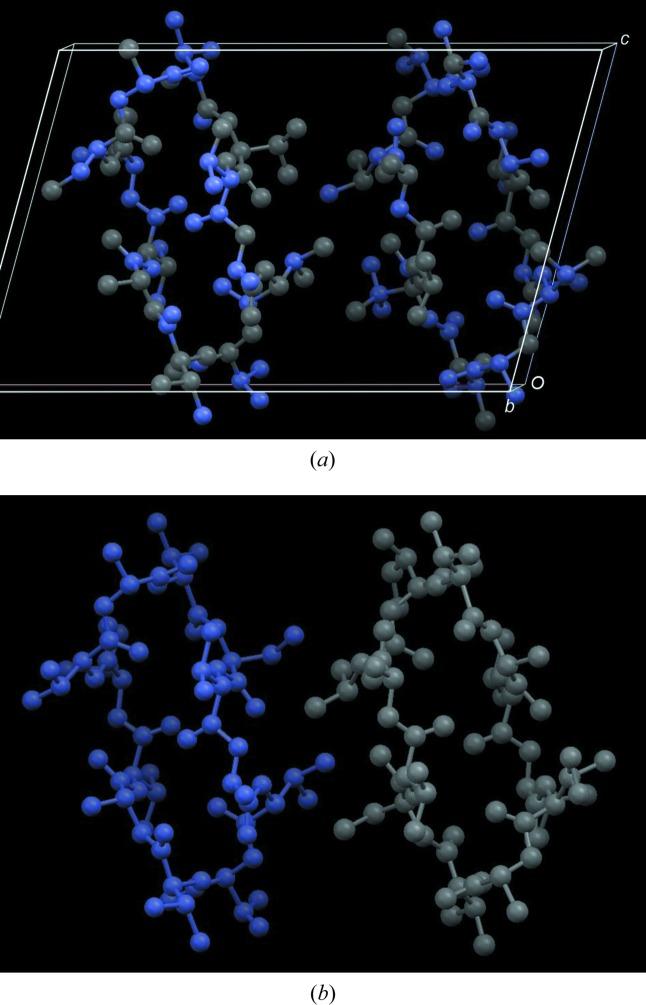
Application of the extended *S*-FFT phase refinement algorithm to intensities of TVAL calculated (*a*) with 50% randomly assigned negative scattering factors and (*b*) with all atomic scattering factors of one of the two symmetry-independent molecules made negative. Atoms with negative refined densities are depicted in grey. The peak search was performed on the Fourier map computed with the phases from the extended *S*-FFT.

Some first applications of the *S*-TF algorithm to non-positive definite density functions in difference structures and in reconstructed surfaces (by using in-plane X-ray diffraction data) can be found in Rius *et al.* (1996 ▶) and Pedio *et al.* (2000 ▶), respectively. However since, for these particular applications, the *S*-FFT algorithm is much simpler and more accurate (all phase invariants are implicitly taken into account), only *S*-FFT is considered here. In all situations so far discussed, it has been assumed that ρ is positive definite, so that *G*, as given by equation (4), corresponds to the amplitude of the squared structure ρ^2^. However, there are certain situations where positivity of ρ is violated. Neutron diffraction data from compounds with negative scatterers are typical cases (Table 3 ▶). In such cases, the corresponding nuclear density function (still designated by ρ) consists of positive and negative scatterers, so that the *G* values derived from equation (4) are no longer the s.f. amplitudes of ρ^2^ but of the so-called ‘squared-shape structure’, in which the atomic peaks have the shape they have in ρ^2^ but preserve the signs they have in ρ. As was shown by Rius & Frontera (2009 ▶), the *S*-FFT algorithm can cope with non-positive definite ρ by simply introducing in equation (13) an *m* mask calculated according to the following scheme
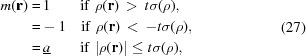
where *t* ≃ 2.5, and a is a random value between −1 and 1. The introduction of *m* into equation (13) yields the extended ***Q*** values, *i.e.*

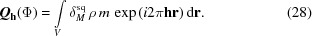
The viability of the algorithm was checked thoroughly with calculated data sets from organic compounds. Fig. 4 ▶ reproduces the Fourier map obtained by processing the intensity data of TVAL (triclinic modification of valinomycin) with the extended *S*-FFT (Karle, 1975 ▶; Smith *et al.*, 1975 ▶).

From the tests performed on a variety of organic compounds it was concluded that the extended *S*-FFT algorithm has a lower convergence rate than the unextended *S*-FFT (approximately two to three times for the studied test cases), so that the number of refinement cycles has to be increased. This is the price that the extended form has to pay for not including the positivity (or, better, the equal-sign) constraint.

For inorganic compounds no significant difference in convergence speed was detected. A perovskite-related compound containing the strong negative neutron scatterer Mn illustrates how the extended *S*-FFT works (Table 3 ▶). The intensities used in the calculations were extracted from the observed powder diffraction pattern by redistributing the global intensities of the overlapping peaks according to the calculated individual intensities (Frontera *et al.*, 2004 ▶). The success rate is three from a total of 25 trials (Rius & Frontera, 2008 ▶).

Another important situation where negative peaks appear in the Fourier map is in the solution of difference structures. An example of this type of application can be found in Rius & Frontera (2008 ▶).

## 3. Cluster-based Patterson-function direct methods for powder data   

### 3.1. Definition of atomic, experimental and effective resolutions   

**Figure 5:**
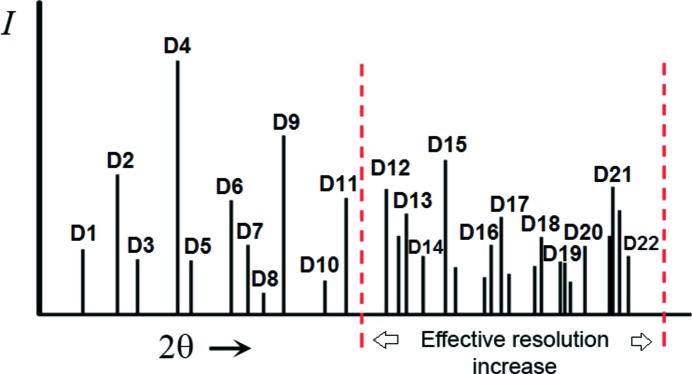
Schematic powder pattern divided into clusters of unresolved reflections. In contrast with traditional DM which only use resolved reflections (low effective resolution), cluster-based DM can process the information of the high-angle region of the pattern. In this way the effective resolution approaches the experimental one.

In contrast with other crystal structure determination methods, the experimental information used by DM is generally limited to the set of measured intensities. This is why it is very important that the data set is almost complete and atomic resolution is reached (only then will the atomic peaks show up clearly separated in the Fourier map). The experimental resolution of a powder pattern is defined by the 2θ value beyond which no more diffraction peaks appear. It is normally expressed in terms of the corresponding *d*-spacing value (*d*_min_). The experimental resolution depends mainly on the crystallinity of the material, *e.g.* materials with small domain sizes have broad diffraction peaks, so that peaks and background are difficult to separate at high 2θ. Also important for the application of DM to PD is the effective resolution concept (Fig. 5 ▶). Since traditional DM use only the intensities from resolved reflections (which are highly dependent on the amount of peak overlap), the pattern region with useful information is reduced. The *d*-spacing corresponding to the upper 2θ limit of this region gives the effective resolution (*d*_eff_). Very often the effective resolution is much less than the experimental one, which hampers the successful application of DM. However, if DM are modified in such a way that clusters of intensities can be treated, the effective resolution of the pattern increases and *d*_eff_ and *d*_min_ become more similar. The introduction of ‘model-free pattern matching’ greatly facilitated the partition of powder patterns into sequences of cluster intensities (Pawley, 1981 ▶; Le Bail *et al.*, 1988 ▶). The inclusion of the cluster information in the structure determination process yields better resolved peaks in the intermediate Fourier syntheses. Summarizing, in the same way that the Rietveld method allows one to take advantage of the whole experimental resolution of the powder pattern during the refinement, cluster-based DM allow one to increase the effective resolution during the solution process, so that it comes much closer to the experimental one.

### 3.2. The cluster-based *S*_*P*_ function for PD   

When PFDM are applied to powder data, the smallest unit of intensity information is the total intensity of each group of unresolved reflections (cluster). The two quantities that specify an arbitrary *j* cluster are:
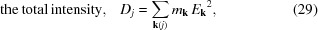


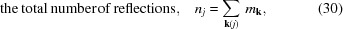
where *m* are the multiplicities of all symmetry-independent reflections. In view of equations (29) and (30), if **H** is an arbitrary reflection of this cluster, the equidistributed intensity for **H** is
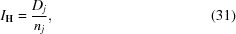
so that 〈*I*〉, its average taken over all reflections, is equal to 〈*E* ^2^〉 = 1.

**Figure 6:**
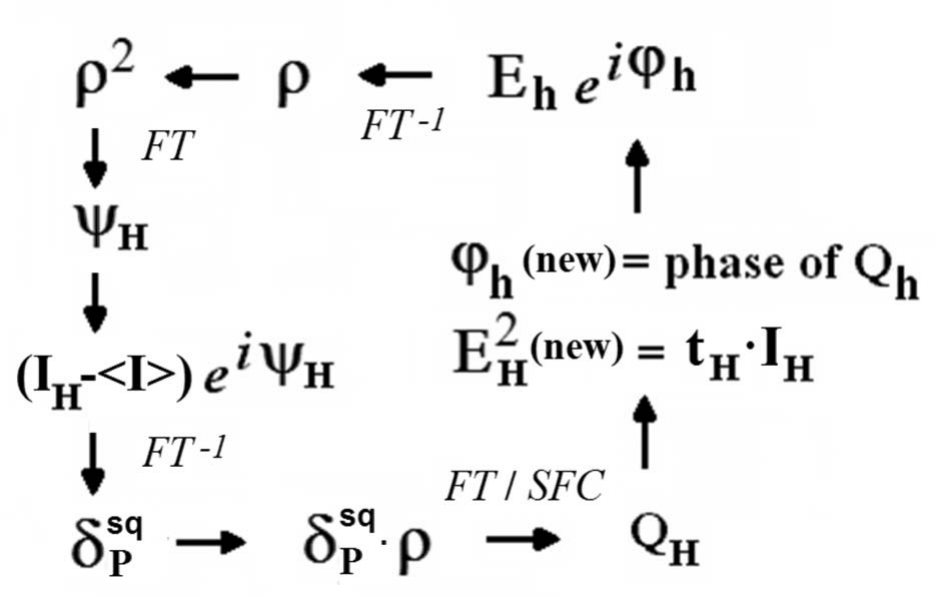
Iterative *S*_*P*_-FFT phase refinement procedure for powder data. Only the differences with respect to the already described *S*-FFT algorithm are indicated (Fig. 3 ▶). Initial phase values (upper right corner) are combined with weighted experimental and extrapolated amplitudes to give the initial ρ values (upper right corner). The Fourier coefficients of the 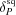 synthesis are obtained by combining the experimental (*I* **_H_** − 〈*I*〉) intensities with the ψ phases. The new structure factor estimates are alternatively obtained either by Fourier transforming δ·ρ (FT) or directly from the *N* top-ranked Fourier peaks (SFC) of δ·ρ. For overlapped reflections, 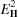 values are updated every cycle [*t* **_H_** is the intensity redistribution coefficient calculated according to equation (33), which ensures the constancy of the global cluster intensity].

In the cluster-based *S*_*P*_ of equation (18), the observed intensities for overlapping reflections are simply their equidistributed values (Rius, 2011 ▶). This is the best approximation to the experimental Patterson function. Notice also that the origin peak can be removed exactly. The refinement of the Φ subset of phases (strong reflections) is achieved by maximizing *S*_*P*_ with the *S*-FFT algorithm. Φ is updated from cycle to cycle and at the end of each trial the cluster-based figure of merit
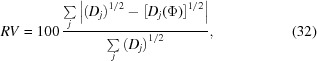
is computed. The solution with the smallest *RV* value is taken as the correct one. To handle PD data, the following modifications in the *S* -FFT phase refinement algorithm are necessary (Fig. 6 ▶):

(i) The coefficients (*G* − 〈*G*〉) in stage 2 must be replaced by the (*G* ^2^ − 〈*G* ^2^〉) ones, so that stage 2 in §2.3.2 becomes ψ + observed (*G* ^2^ − 〈*G* ^2^〉) → 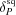 → 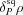 → ϕ_new_.

(ii) Those ρ values below *t*σ(ρ) are made zero.

(iii) The calculation of the Fourier coefficients (***Q***) of the product function 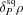 is performed either by direct Fourier transformation (FT) or by structure factor calculation (SFC) from the *N* highest peaks in 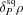 (Fig. 6 ▶). The periodic calculation of the structure factors from the *N* peaks is carried out to ensure the fulfilment of the randomness condition. (With single-crystal data this step is normally not necessary).

(iv) The intensities of overlapping reflections are re­distributed according to
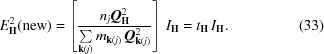
The physical meaning of the cluster-based *S*_*P*_ function can be best understood by writing it in terms of the cluster intensities. In view of the proportionality between the integrals of equations (17) and (24), *S*_*P*_ in equation (18) may be assumed to be proportional to
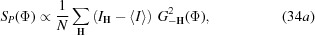
so that by equation (31) it follows
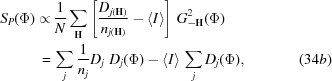
and *S*_*P*_ essentially corresponds to the sum of the products of the observed and calculated cluster intensities, divided by the number of reflections contributing to each cluster. As long as Φ fulfills the general properties of the electron-density distribution (positivity, randomness, atomicity), the second sum in equation (34*b*) can be regarded as constant during the phase refinement.

### 3.3. Examples of application of the cluster-based *S*-FFT algorithm   

**Figure 7:**
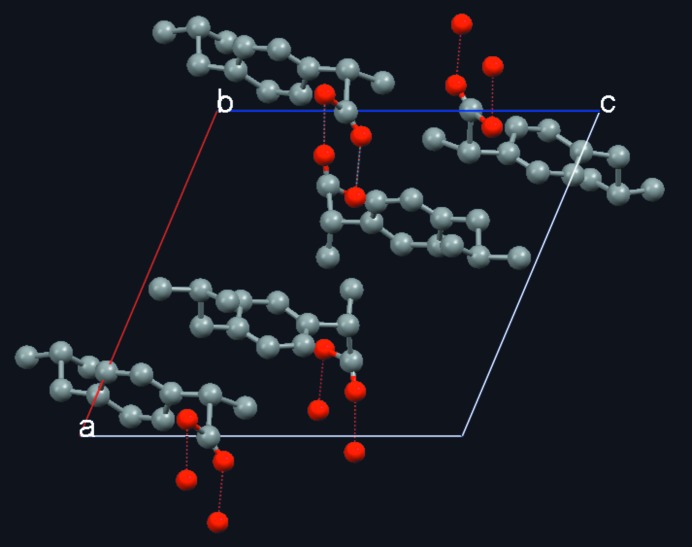
Perspective view, along the *b* axis, of the monoclinic unit cell of ibuprofen (Freer *et al.*, 1993 ▶), with atomic positions taken directly from the cluster-based DM Fourier map (*a* = 12.46, *b* = 8.03, *c* = 13.53 Å, β = 112.95°, *V* = 1246 Å^3^). There are two symmetry-independent molecules which form a dimer. Image created with *Mercury* (Macrae *et al.*, 2008 ▶).

**Figure 8:**
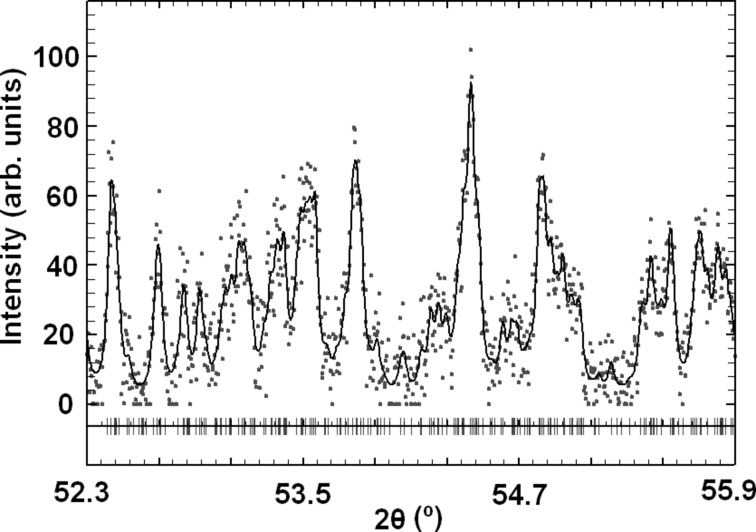
Whole-profile refinement without a structural model for intensity extraction; portion of the ibuprofen powder pattern centred at *d* ≃ 1.10 Å to show peak overlap. Only the pattern information above *d*_min_ = 1.10 Å (< 2θ ≃ 54°) was processed by *XLENS_PD6* (Rius, 2011 ▶). Plot created with *WinPLOTR* (Roisnel & Rodríguez-Carvajal, 2001 ▶). Widths of Lorentzian profiles in full width at half-maximum (FWHM) are 0.014, 0.023, 0.031 and 0.040° for respective *d* spacings > 10, 2.5, 1.5 and 1.1 Å; overlap criterion < 0.5 FWHM.

Retrospectively, the development of the cluster-based *S*-FFT algorithm was greatly facilitated by the release of some high-quality PD patterns of organic compounds collected by Dr Gozzo for the Summer School on ‘Structure Determination from PD Data’ organized at the Swiss Light Source in 2008. These patterns had been measured with the novel Mythen-II microstrip one-dimensional detector (Schmitt *et al.*, 2004 ▶). For a detailed study of the *S*_*P*_ function with powder data, the pattern of (*S*)-(+)-ibuprofen was selected (Freer *et al.*, 1993 ▶). The monoclinic unit cell contains two symmetry-independent molecules, giving rise to a cyclic hydrogen-bonded dimer with the formula C_26_H_16_O_4_ (Fig. 7 ▶). The intensities were extracted by pattern matching using *DAJUST* (*d*_min_ = 1.10 Å for λ = 1.0 Å) (Vallcorba *et al.*, 2012 ▶). Details of the peak profiles are given in Fig. 8 ▶. The extracted cluster intensities (total number of reflections is 1009) were processed by the *XLENS_PD6* program, which has the cluster-based *S*-FFT implemented (downloadable from http://departments.icmab.es/crystallography/software). During the phase refinement, chemical constraints were applied every second refinement cycle. Seven trials out of 25 were successful (50 cycles per trial). All correct solutions developed the complete structural model. Some relevant details of the model extracted from the Fourier map are listed in Table 4 ▶ (Rius, 2011 ▶).

In spite of being relatively new, the cluster-based *S*-FFT algorithm has already solved some rather difficult unknown crystal structures from conventional laboratory PD data, *e.g.* those of the highly hydrated minerals sanjuanite, Al_2_(PO_4_)(SO_4_)(OH)·9H_2_O, *Z* = 4, space group *P*2_1_/*n* (Colombo *et al.*, 2011 ▶), and sarmientite, Fe_2_^3+^(AsO_4_)(SO_4_)(OH)·5H_2_O, *Z* = 4, space group *P*2_1_/*n, V* = 1156 Å^3^ (Colombo *et al.*, 2014 ▶), or the triclinic crystal structure of a new partially deprotonated mixed-valence manganese(II,III) hydroxide arsenate related to sarkinite (de Pedro *et al.*, 2012 ▶). Cluster-based *S*-FFT has also determined the frameworks of hybrid materials like calcium hydroxyphosphonoacetates (Colodrero *et al.*, 2011 ▶), magnesium tetraphosphonate (Colodrero *et al.*, 2012 ▶) or calcium glyceroxide, an active phase for biodiesel production under heterogeneous catalysis (León-Reina *et al.*, 2013 ▶). Due to the presence of the organic part, synchrotron radiation is preferred for hybrid materials. This normally gives higher experimental resolution (compared with laboratory data), which helps to develop the complete crystal structure model at the end of the phase refinement stage.

## 4. The δ recycling method   

### 4.1. The calculated ρ (based on the δ function)   

The δ recycling method is an extremely simple phasing method. It is based on a function δ_*M*_, which is the convolution of *P*′ (of the true structure) with a phase synthesis. Experimentally, δ_*M*_ is computed with the Fourier syntheses
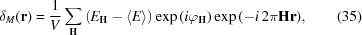
and consists of maxima at the atomic positions and noise in between. According to Rius (2012*a* ▶), the strength of δ_*M*_ at the **r** *_k_* atomic positions can be approximated, for an equal atom structure, by
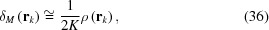
with 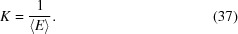
Independently, the variance of δ_*M*_ only depends on the amplitudes and is given by
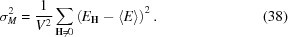
The fact that 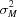 is independent of the phase estimates allows one to fix a threshold value before the structure is solved (Rius, 2012*a* ▶). In practice, the threshold value Δ = *t*δ_*M*_ with *t* ≃ 2.5 works well for eliminating noise. In this way an *m* mask can be created, which will be 0 or 1 depending on whether the corresponding δ_*M*_ value is below or above Δ. By multiplying δ_*M*_ by this mask and considering equations (36) and (37), the desired approximation to ρ is obtained
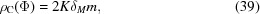
which must be always positive and uses the known *E* magnitudes (Rius, 2012*b* ▶).

### 4.2. The phasing residual and the algorithm   

**Figure 9:**
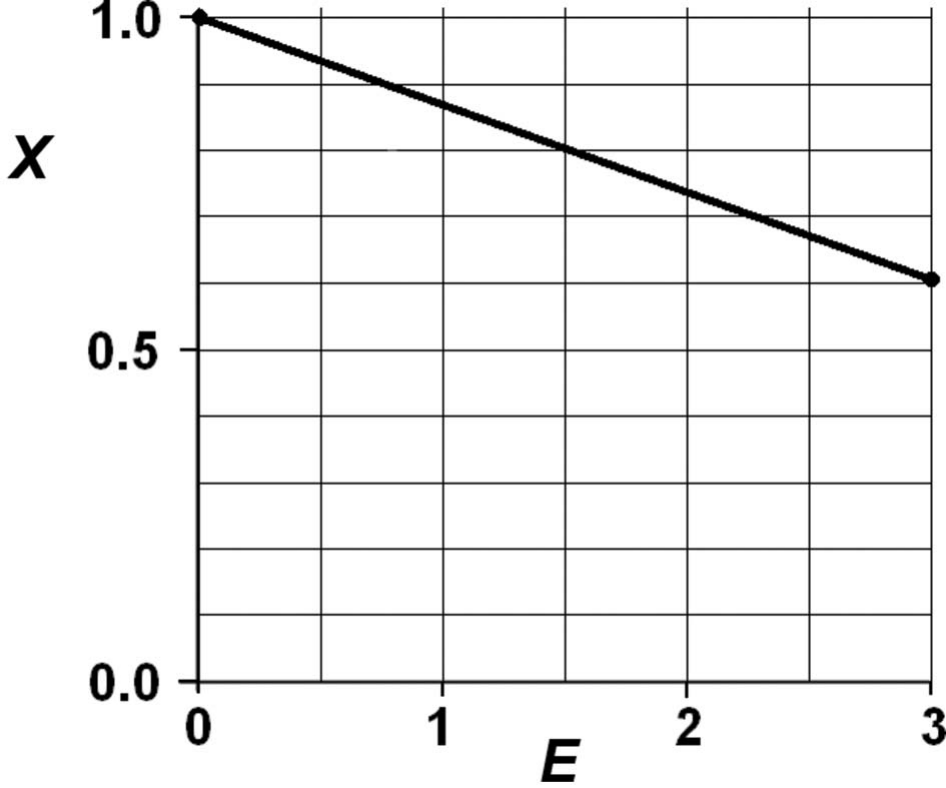
Linear dependence of *X* upon *E* in equation (45) for a *P*1 equal-atom crystal structure.

**Figure 10:**
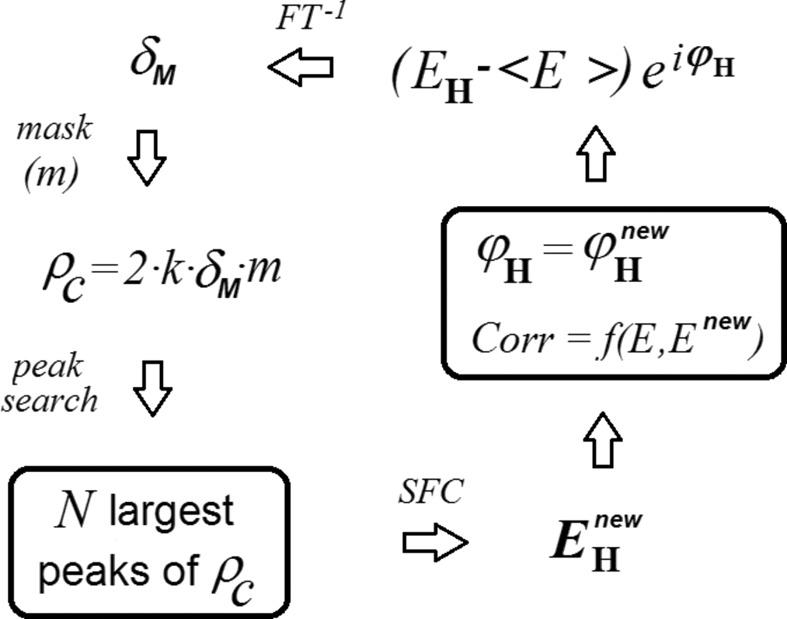
Schematic description of the δ_*M*_ recycling phasing procedure. Starting random phase values are fed in at the upper right corner. For PED/ADT intensity data, iteration stops when a preset number of cycles is reached. The meaning of the different symbols is explained in the text (SFC = structure factor calculation). For δ_*P*_ recycling, the *E* − 〈*E*〉 coefficients are replaced by *E* ^2^ − 〈*E* ^2^〉 and the *M* subscript by *P*.

If ρ(**r**) represents a positive definite density function of the crystal, *e.g.* the electron density or the electrostatic potential (in this second case only for structure solution purposes), it will be assumed that the condition
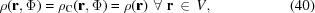
is only fulfilled for the true Φ values. The discrepancy between ρ(**r**,Φ) and ρ_C_(**r**,Φ) can be measured through the residual
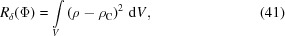
extended over the whole unit cell of volume *V*, where for clarity the **r** and Φ symbols have been omitted in the integrand. By working out the squared binomial, and since the integral of ρ^2^ over the unit cell is phase-independent (it corresponds to the value of the Patterson function at the origin and is equal to 1/*V* ∑**_H_** *E* **_H_** ^2^), minimizing *R*_δ_ is equivalent to maximizing the integral
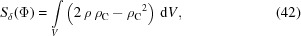
which in view of equations (37) and (39), and because *m* = *m* ^2^, reduces, after some algebraic manipulation, to
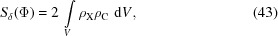
wherein ρ_X_ corresponds to
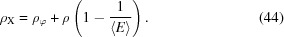
Here, ρ_ϕ_ denotes the phase synthesis, *i.e.* a Fourier synthesis with the same phases as ρ but with constant amplitudes (in this case unity). In view of this, it follows from equation (44) that the Fourier coefficients of ρ_X_ are
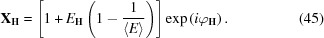
The dependence of the modulus of **X** on the amplitude *E* is of a linear type (Fig. 9 ▶). By expressing ρ_X_ in equation (44) as a Fourier synthesis, equation (43) transforms into
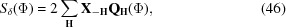
with
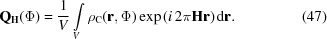
Equation (46) is formally equivalent to equation (11), except for the fact that the summation extends over all **H** reflections, not just the strongest ones (Rius, 2012*b* ▶). Consequently, the new phase estimates can also be derived by applying a tangent formula, namely
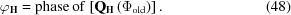
The general scheme of the δ recycling phasing procedure is described in Fig. 10 ▶. As indicated by the structure factor calculation (SFC), the 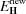 structure factors are computed from the *N* largest peaks found in ρ_C_ [equation (39)]. The new Φ set is then used to update δ_*M*_. This procedure is applied cyclically until convergence is reached. Convergence is controlled by measuring the correlation Corr between the experimental *E* and the updated *E*_new_ with the expression
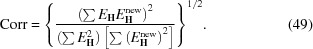



### 4.3. Application to ED tomography data   

Frequently, natural and synthetic phases only appear as sub-micrometric crystals, too small for collecting single-crystal X-ray data even with synchrotron radiation. Normally, structural information from these phases is obtained from PD, which combines easy sample preparation (also under non-ambient conditions) with fast acquisition systems and sophisticated analytical methods. Nevertheless, PD suffers from various limitations which may be caused by the sample [(i) sufficient sample must be available; (ii) the sample must be an almost pure phase; (iii) for nanocrystals, peak broadening due to the particle size reduces the effective data resolution range] and/or by the crystal structure itself [(i) indexing of unit cells with long axes is not always trivial; (ii) systematic overlap is present in high-symmetry space groups, especially in cubic ones; (iii) accidental overlap may be severe for low-symmetry space groups]. In addition, identification of the space group for crystalline phases affected by pseudo-symmetry can be problematic even for good PD data [see, for example, Birkel *et al.* (2010 ▶) and Rozhdestvenskaya *et al.* (2010 ▶)]. The main advantage of electron diffraction (ED) is the ability to collect single-crystal data from nanometric volumes. This is possible because electrons can be deflected and focused in quasi-parallel probes with a diameter of 10–30 nm and because the interaction with matter for electrons is much stronger than for X-rays, allowing a good signal-to-noise ratio even for diffraction from nanovolumes of crystalline material. Two of the principal problems of ED, *i.e.* dynamic effects and incomplete data sets, are minimized by measuring off-zone. This is the basis of the automated diffraction tomography (ADT) data collection strategy (Kolb *et al.*, 2007 ▶, 2008 ▶). In ADT, the ED patterns are acquired by rotating around an arbitrary tilt axis (not corresponding to a specific crystallographic orientation) in sequential steps of 1° within the full tilt range of the microscope. The physical limit affecting the sample rotation gives rise to incomplete data sets, *i.e.* to a missing wedge. The precession ED technique (PED) is used to integrate the intensity between steps. Recently, an alternative technique called rotation ED (RED) has been introduced for this purpose (Zhang *et al.*, 2010 ▶). Of course, there are also disadvantages with ED. For certain compounds, radiation damage is still a limiting problem. In general, organic and hybrid materials are more beam-sensitive than inorganic materials. The application of δ recycling to PED/ADT intensities from inorganic materials was recently analyzed by Rius *et al.* (2013 ▶) with some interesting results: (i) scaling with the Wilson plot procedure is accurate; (ii) δ recycling is particularly robust against missing data; (iii) unlike X-rays, where Corr [equation (49)] clearly discriminates the correct solution, the final Corr values with PED/ADT data tend to be similar for correct and wrong solutions. To circumvent this difficulty, the δ recycling phasing stage always terminates when a preset number of cycles is reached, and continues with conventional Fourier recycling methods. Convergence during Fourier recycling is controlled by the *R*_CC_ residual,
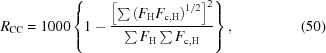
which is free from scaling factors. *R*_CC_ is always a very reliable figure of merit and for X-rays values between 5–30 indicate correct solutions; for PED/ADT data, essentially correct solutions are found between 15 and 60, although *R*_CC_ values up to 80 can be reached, especially if the data are affected by large thickness variations and/or by residual dynamic scattering, if missing organic parts of the structure are not included in the calculation of the intensities, or if the measured data fail to produce well shaped peaks in the Fourier map.

**Figure 11:**
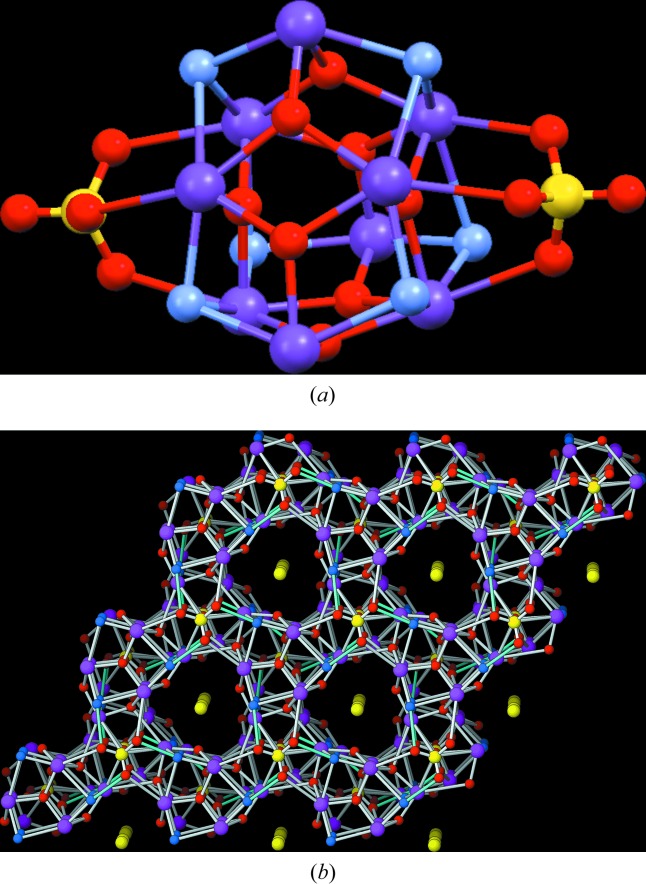
Crystal structure of the porous Bi sulfate mineral of the Alfenza mine (Crodo, Italy) solved by δ recycling from PED/ADT data (Capitani *et al.*, 2014 ▶). Crystal data: *a* = 9.5 (2), *c* = 15.4 (3) Å, *V* = 1200 Å^3^, space group 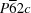. (*a*) Bi sulfate cluster with the formula [Bi_8.18_Te_0.82_(OH)_6_O_8_(SO_4_)_2_]^0.91+^ (hydroxyl groups are at the corners and the long cluster dimension is along *c*). (*b*) Self-assembly of Bi sulfate clusters gives rise to a porous framework, which is held together by (i) hydrogen bonds between hydroxyl groups and sulfate ligands; and (ii) weak Bi—O bonds (the coordination polyhedra of both symmetry-independent Bi^3+^ cations are pentagonal bipyramids). The disulfide anions in the one-dimensional channels (appearing in the Fourier synthesis) help to stabilize the structure.

It goes without saying that the possibility of solving crystal structures from phases only detected by TEM is very important in many research fields. For example, it is expected that many new mineralogical species can be found. In a recent collaboration with Kolb’s group at the University of Mainz, δ recycling has solved, from PED/ATD data, the crystal structure of a new porous Bi sulfate mineral appearing only as a tiny crystalline fragment (∼0.15 × 0.15 × 0.2 µm) displaying no net cleavage planes (Capitani *et al.*, 2014 ▶). The crystal structure is hexagonal and the unit-cell content is [Bi_8.18_Te_0.82_(OH)_6_O_8_(SO_4_)_2_]^0.91+^·0.91S_2_^−^ with *Z* = 2 (Fig. 11 ▶). Some relevant experimental details are: *d*_min_ = 1.0 Å, number of measured (unique) reflections = 2748 (452), *R*_equiv_ = 23.57, data completeness = 100%; λ = 0.0197 Å, *T* = 93 K. The structure model obtained from δ recycling was complete and can be described as a self-assemblage of Bi clusters giving rise to a one-dimensional porous material with the disulfide anions inside the channels. Figures of merit for the last refinement cycle are *R*_1_ = 0.2173 for 332 *F*_obs_ > 4σ(*F*_obs_) and 0.2373 for all 452 data, *i.e.* of the same order as the *R* value between symmetry-equivalent reflections (*SHELX97*; Sheldrick, 2008 ▶). Finally, it is worth mentioning that the O atoms could be located in the presence of the extremely heavy Bi atoms (*Z* = 83), a consequence of the slower scattering-power increase with atomic number compared with X-rays.

## 5. Conclusions   

Currently, the application of DM has reached maturity. This means that, for an ideal single-crystal intensity data set, phasing is a rather straightforward process. However, the situation changes for inaccurate or incomplete data sets, a circumstance which occurs with increasing frequency in materials science, especially when small crystalline volumes are being analyzed. To deal with these situations, not only robust but also simple DM procedures are required which can process, in a unified manner, partial information coming from different sources, *e.g.* transmission microdiffraction, electron diffraction, powder diffraction, grazing-incidence diffraction. Due to their simplicity, PFDM are ideal candidates for such types of applications which also benefit from rapidly evolving instrumental capabilities.

## Figures and Tables

**Table 1:** Comparison of success rates (%) for two tangent formula algorithms based on phase relationships (*a*) *S*-TF with a varying number of strong reflections. (*b*) Traditional TF (Karle & Hauptman, 1956 ▶), complemented with the most reliable negative quartets (values taken from Sheldrick, 1990 ▶). *S*-TF is clearly superior, especially for space groups having the origin floating at least in one direction.

Code	Reference	Space group	*N*	(*a*)	(*b*)
BHAT	Bhat & Ammon (1990 ▶)	*Pc*	200	42.5–71.5	0.26
HOPS	Jones *et al.* (1992 ▶)	*R*3	1000	9.6–13.7	0.56
MBH2	Poyser *et al.* (1986 ▶)	*P*1	300	39.0–67.6	4.7
LOG	Jones *et al.* (1980 ▶)	*P*2_1_2_1_2_1_	108	5.3–6.2	2.1
SUOA	Oliver & Strickland (1984 ▶)	*P*2_1_2_1_2_1_	188	0.4–1.8	0.06
PEP1	Antel *et al.* (1990 ▶)	*P*2_1_2_1_2_1_	340	0.08–0.2	0.01

**Table 2:** Comparison of the *S*-FFT and *S*-TF phase refinement algorithms for different compounds (data resolution limit in the 0.85–1.04 Å *d* spacing interval) As expected, both *S*-FFT and *S*-TF algorithms yield similar success ratios, although *S*-TF is much more efficient in computing time (for small molecules).

Code	Unit-cell content	Space group	No. of refined phases	No. of solutions *S*-FFT/*S*-TF	No. of trials (cycles)
PGE2^*a*^	C_20_H_32_O_5_	*P*1	161	21/25	25 (11–37)
MBH2^*b*^	C_45_H_72_O_9_	*P*1	580	24/24	25 (15–70)
TVAL^*c*^	C_108_H_180_N_12_O_36_	*P*1	1043	23/25	25 (19–54)
NEWQB^*d*^	C_96_H_80_N_8_O_20_	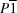 as *P*1	663	24/25	25 (21–60)
GOLDMAN2^*e*^	C_224_H_128_	*Cc*	607	25/25	25 (16–45)
BHAT^*f*^	C_20_H_16_N_20_O_36_F_8_	*Pc*	285	11/16	25 (24–49)
HOV1^*g*^	Pr_56_Ni_32_Si_36_	*C*2/*m* as *Cm*	518	22/25	25 (14–67)
MUNICH1^*h*^	C_160_H_128_	*C*2	352	2/2	50 (18–55)
BIH^*i*^	C_56_B_36_H_152_O_12_N_4_S_8_	*P*2_1_/*c* as *Pc*	661	25/25	25 (10–20)
CORTISON^*j*^	C_84_H_112_O_20_	*P*2_1_2_1_2_1_	247	6/14	50 (17–47)
BNA^*k*^	C_40_B_36_H_100_O_12_S_8_Na_4_	*Pnma, P*2_1_2_1_2_1_	303	5/7	25 (9–12)
WINTER2^*l*^	C_110_H_178_N_22_O_32_Cl_12_	*P*2_1_	1045	6/1	25 (30–53)
TOTC^*m*^	C_198_H_216_O_36_	*P*6_1_	301	20/25	25 (17–51)
TUR10^*n*^	C_180_H_288_O_24_	*P*6_3_22	160	6/8	50 (20 fixed)
BED^*o*^	C_208_H_208_N_32_O_32_	*I*4	285	8/8	25 (18–50)
ALFA1^*p*^	C_328_O_110_N_65_H_500_	*P*1	3772	3/†	360 (88–179)

References: (*a*) DeTitta *et al.* (1980 ▶); (*b*) Poyser *et al.* (1986 ▶); (*c*) Smith *et al.* (1975 ▶); (*d*) Grigg *et al.* (1978 ▶); (*e*) Irngartinger *et al.* (1981 ▶); (*f*) Bhat & Ammon (1990 ▶); (*g*) Hovestreydt *et al.* (1983 ▶); (*h*) Szeimies-Seebach *et al.* (1978 ▶); (*i*) Teixidor *et al.* (1991 ▶); (*j*) Declercq *et al.* (1972 ▶); (*k*) Teixidor *et al.* (1990 ▶); (*l*) Butters *et al.* (1981 ▶); (*m*) Williams & Lawton (1975 ▶); (*n*) Braekman *et al.* (1981 ▶); (*o*) Sheldrick *et al.* (1978 ▶); (*p*) Privé *et al.* (1999 ▶).

† The *S*-TF refinement with the same control parameters was not carried out due to the large number of triplets generated.

**Table 3:** Application of the extended *S*-FFT algorithm to neutron diffraction data of the perovskite-related compound with unit formula (Bi_0.75_Sr_0.25_)MnO_3_ Crystal data: *a* = 5.499, *b* = 7.770, *c* = 5.542 Å, space group *Imma, Z* = 4. Phase refinement gives a large negative peak for Mn in the *E* map, as expected for a negative scatterer (Fermi lengths for Bi, Sr, Mn and O are, respectively, 0.853, 0.702, −0.373 and 0.580).

Atom	Relative height	*x*/*a*	*y*/*b*	*z*/*c*	Site
Bi,Sr	1000		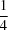	0.9974	4*e*
Mn	−579	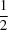			4*b*
O1	474	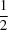	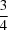	0.0501	4*e*
O2	409	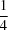	0.0275	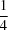	8*g*

**Table 4:** Bond lengths (Å) obtained by applying the cluster-based *S*-FFT algorithm to powder diffraction (PD) data and by least-squares refinement from single-crystal (SC) data Bonds have been grouped into types: (i) average C—C bond lengths from PD and SC (the former are longer by a factor of 1.035, which may be attributed to the non-inclusion of H atoms in DM); (ii) single and double C—O bonds are correctly assigned with similar average lengths, 1.30 (PD) and 1.27 Å (SC); (iii) distances between hydrogen-bonded O atoms are practically coincident.

Bond type	PD	SC
C—C (single bond) (14×)	1.56 (9)	1.51 (3)
C—C (phenyl ring) (12×)	1.43 (8)	1.38 (2)
C—O (≃ single bond) (2×)	1.50 (3)	1.32 (1)
C—O (≃ double bond) (2×)	1.09 (6)	1.21 (1)
O⋯O (hydrogen bonds) (2×)	2.66 (1)	2.65 (1)

## Appendix A Simplification of the *R*_*M*_(Φ) expression

Written in terms of the Fourier coefficients of *M*′, the integral of equation (7) is equivalent to

If *K*_M_ = Σ_**H**_(*G*_**H**_ − *G*)^2^, then

By transforming the sums into averages (*N* **_H_** = number of reflections), the last two summations reduce to

Finally, replacement of the last two last sums in equation (52) by equation (53) leads to the simplified *R*_*M*_(Φ) expression
